# Effect of Hyaluronic Acid Compared to Platelet-Rich Plasma as Adjuvants to Bone Marrow Mesenchymal Stem Cell Treatment of Knee Osteoarthritis: Analysis from Two Clinical Trials

**DOI:** 10.3390/diagnostics15030309

**Published:** 2025-01-28

**Authors:** José María Lamo-Espinosa, Álvaro Suárez-López del Amo, Jorge María Núñez-Córdoba, Juan F. Blanco, Mikel Sánchez, Victoria Moreno, Marta Cabrera, Froilán Granero-Moltó, Emma Muiños, Manuel M. Mazo, Íñigo Crespo-Cullell, Gonzalo Mora, Diego Delgado, Orlando Pompei-Fernández, Jesús Dámaso Aquerreta, María Vitoria Sola, Andrés Valentí-Azcárate, Enrique J. Andreu, Miriam López-Parra, Eva M. Villarón, Juan Ramón Valentí-Nin, Fermín Sánchez-Guijo, Felipe Prósper

**Affiliations:** 1 Department of Orthopaedic Surgery and Traumatology, Clínica Universidad de Navarra, 36 Pío XII Avenue, 31008 Pamplona, Spain; 2 Cell Therapy Area, Clínica Universidad de Navarra, Regenerative Medicine Program, Cima Universidad de Navarra and Instituto de Investigación Sanitaria de Navarra (Idisna), 31008 Pamplona, Spain; 3 Division of Biostatistics, Research Support Service, Central Clinical Trials Unit, Clínica Universidad de Navarra, 31008 Pamplona, Spain; 4 Department of Preventive Medicine and Public Health, Medical School, University of Navarra, 31008 Pamplona, Spain; 5 Department of Orthopaedic Surgery and Traumatology, University Hospital of Salamanca—Instituto de Investigación Biomédica de Slamanca (IBSAL), University of Salamanca, 37008 Salamanca, Spain; 6 Arthroscopic Surgery Unit, Hospital Vithas Vitoria, 01008 Vitoria-Gasteiz, Spain; 7 Advanced Biological Therapy Unit, Hospital Vithas Vitoria, 01008 Vitoria-Gasteiz, Spain; 8 Department of Radiology, Clínica Universidad de Navarra, 31008 Pamplona, Spain; 9 Department of Hematology, University Hospital of Salamanca—Instituto de Investigación Biomédica de Slamanca (IBSAL), University of Salamanca, 37008 Salamanca, Spain; 10 Department of Hematology and Cell Therapy, Clínica Universidad de Navarra, 36 Pío XII Avenue, 31008 Pamplona, Spain

**Keywords:** platelet-rich plasma, hyaluronic acid, cell therapy, mesenchymal stem cells, osteoarthritis

## Abstract

**Background:** Bone marrow mesenchymal stem cell (BM-MSC) therapy has emerged as a safe and feasible treatment option for patients with knee osteoarthritis (OA). However, the role of adjuvants remains unclear. Our aim was to evaluate the clinical and radiological effects of hyaluronic acid (HA) in comparison to platelet-rich plasma (PRP) as adjuvants to 100 × 10^6^ BM-MSCs in the treatment of knee OA. **Methods:** We used data from two randomized, parallel-group and controlled clinical trials which tested the efficacy of BM-MSC, previously published in 2016 (Clinical Trials.gov identifier NCT02123368, Nº EudraCT: 2009-017624-72) and 2020 (Clinical Trials.gov identifier NCT02365142. Nº EudraCT: 2011-006036-23). **Results**: Of the 34 patients included in the study, 24 had received 100 × 10^6^ BM-MSCs plus PRP and 10 had received 100 × 10^6^ BM-MSCs plus HA. On average, BM-MSC plus HA showed a higher improvement in VAS for pain [β-coefficient: −1.25; 95% confidence interval (95% CI):−2.20 to −0.30) than BM-MSC plus PRP (*p* = 0.01). We also observed that BM-MSC plus HA showed a greater improvement in all the WOMAC subscales scores and in the WOMAC overall score, compared to BM-MSC plus PRP, although these differences were not statistically significant. The Whole-Organ Magnetic Resonance Imaging Score (WORMS) at 12 months was more beneficial with 100 × 10^6^ BM-MSCs plus HA (β-coefficient: −12.61; 95% CI: −19.71, −5.52) than with BM-MSC plus PRP (*p* = 0.001). **Conclusions:** The clinical and radiological outcomes after BM-MSC therapy for knee OA could differ according to the adjuvant employed. HA showed greater clinical effectiveness and fewer instances of articular degeneration than PRP as an adjuvant.

## 1. Introduction

The intra-articular treatment of knee osteoarthritis (OA) with bone marrow mesenchymal stem cells (BM-MSCs) has been shown to be a safe and feasible procedure in numerous phase I and II clinical trials. However, some doubts about its efficacy, mechanism of action, dosing and use of adjuvants are still unresolved [1,2,3,4,5].

In 2017, AI Caplan promoted changing the meaning of the MSC acronym from Mesenchymal Stem Cells to Medicinal Signaling Cells, in response to the paracrine effect that these cells have and their ability to respond to external stimuli [6]. In that context, the use of an adjuvant could make sense.

Hyaluronic acid (HA) and platelet-rich plasma (PRP) are the most frequent adjuvants used with MSCs in OA clinical trials [2,3,5,7,8]. The use of PRP in the treatment of OA has attracted the most interest in the last three decades [9,10]. Anabolic growth factors (basic FGF, TGF-β1, TGF-β2, EGF, IGF-I, PDGF-AB, PDGF-BB, VEGF) and anti-inflammatory cytokines (IL-1ra, sTNF-R1, sTNF-RII, IL-4, IL-10, IL-13, IFNγ) have been described as components of PRP and have therapeutic potential in the treatment of OA in combination with MSCs [11,12,13]. However, different methods of production and standardization make the interpretation and conclusions on their use difficult [10,14].

Similarly, a debate regarding the clinical use of HA in OA treatment is currently ongoing [15,16,17,18,19,20]. HA is a major component of the extracellular matrix and mediates wound repair, cellular signaling, and morphogenesis [21]. Some authors have emphasized that HA viscosupplementation reduces pain and restores joint function [15,16,17]. In contrast, others report no improvement over the placebo [18,19,20]. In vitro, the interaction between MSC and HA has been previously described, inducing an increase in chondrogenesis, an anti-inflammatory effect, and the retention of MSCs; however, these results have not yet been confirmed in vivo [22].

We previously published two multicenter randomized phase I and II clinical trials [3,5] in which patients were treated with 100 × 10^6^ BM-MSC plus PRP and 100 × 10^6^ BM-MSC plus HA, and those treated were considered OA treatment responders according to the Osteoarthritis Research Society International (OARSI) criteria [23]. The combination of data from both trials offers the unique opportunity to compare the effects of different adjuvants in a cohort comprising similar patients that received the same BM-MSCs dose. Our aim was to evaluate the clinical and radiological effects of HA, in comparison to PRP, as adjuvants to the 100 × 10^6^ BM-MSC treatment of knee OA.

## 2. Materials and Methods

We used data from two multicenter, randomized, parallel-group and active-controlled clinical trials testing the efficacy of BM-MSC therapy for knee OA. The trial by Lamo-Espinosa et al. [3] (Clinical Trials.gov identifier NCT02123368. Nº EudraCT: 2009-017624-72) was a phase I/II clinical trial. This study included 30 patients with knee OA who were randomly assigned to the intra-articular administration of HA alone (*n* = 10), 10 × 10^6^ BM-MSCs plus HA (*n* = 10), or 100 × 10^6^ BM-MSCs plus HA (*n* = 10). The second trial by Lamo-Espinosa et al. [5] (Clinical Trials.gov identifier NCT02365142. Nº EudraCT: 2011-006036-23) was a phase II clinical trial that was designed to include 60 patients with knee OA. These patients were randomly assigned to receive PRP or 100 × 10^6^ BM-MSCs plus PRP. We selected two groups of patients from these two clinical trials who were treated with 100 × 10^6^ BM-MSCs plus HA (Hyalone^®^, Fidia Farmaceutici, Madrid, Spain) (*n* = 10) or 100 × 10^6^ BM-MSCs plus PRP (PRGF^®^) (*n* = 30; 24 patients were included in the final analysis due to the detection of trisomy in the culture, *n* = 1; withdrawal of consent, *n* = 1 and loss of follow-up, *n* = 4) (Supplementary Figure S1). All procedures were approved by the Institutional Review Board of Navarra and the Spanish Agency of Medicines and Medical Devices. All participants provided written informed consent.

The protocols applied in both clinical trials have been published previously in the respective publications [3,5]. Both protocols followed similar steps, except for the age range: 50–80 years in the first clinical trial and 18–80 years in the second. The procedures for cell culture, PRP preparation, HA administration and treatment delivery have been detailed previously [3,5]. The scales used to assess pain (VAS) and function (WOMAC) were evaluated at baseline, 1, 3, 6 and 12 months after treatment. The imaging data (WORMS protocol) were evaluated blindly by a musculoskeletal radiologist [23,24,25].

### 2.1. Cell Culture

BM-MSCs were generated under good manufacturing practice (GMP) conditions following standard operating procedures, as previously described [3].

### 2.2. PRP Preparation

PRP was prepared through a centrifugation process at 580× *g* for 8 min at room temperature. The upper plasma layer, which had a platelet count comparable to peripheral blood, was discarded. Instead, the fraction of plasma located just above the sedimented red blood cells, excluding the buffy coat, was collected in a separate tube. This fraction showed a moderate increase in platelet concentration, containing 2 to 3 times the platelet count of peripheral blood and being free of leukocytes. To activate the platelets, 10% calcium chloride was added at a ratio of 0.05 mL per ml of PRP in the injection room. This protocol was carried out using the PRGF–Endoret system https://bti-biotechnologyinstitute.com/en/solutions/regenerative-medicine/why-endoret-prgf accessed on 23 December 2024 (BTI System II; BTI Biotechnology Institute, Vitoria-Gasteiz, Spain).

### 2.3. Cells, PRP (PRGF^®^) and HA (Hyalone^®^) Injection

The administration of BM-MSCs and PRP was via a lateral patellar approach, as outlined in previous studies [3]. The cell injection procedure was scheduled 3–4 weeks after the iliac crest biopsy. For all patients, the harvested cells were injected within one hour of collection. A 19 G needle was employed to perform two intra-articular injections consecutively. The first injection delivered 100 × 10^6^ BM-MSCs suspended in 3 mL of Ringer’s lactate. Following this, 8 mL of PRP were introduced through the same pathway. Additional doses of 8 mL of PRP were administered in two separate sessions, each spaced one week apart.

In the case of BM-MSCs and HA treatments, 3 mL of BM-MSCs were injected initially, followed by 4 mL of HA (Hyalone^®^) via the same injection route.

### 2.4. Statistical Analysis

The demographic and clinical characteristics of patients were summarized using means with standard deviations, for quantitative variables, and counts with percentages, for qualitative variables. Linear mixed-effects models were used to take dependencies from the data into account. A *p*-value < 0.05 was considered statistically significant. Statistical analyses were performed using Stata 14 (StataCorp. 2015. Stata Statistical Software: Release 14. College Station, TX, USA: StataCorp LP).

## 3. Results

### 3.1. Demographic and Clinical Data

A total of 34 patients were prospectively studied. Twenty-four patients received 100 x10^6^ BM-MSCs plus PRP and 10 patients received 100 × 10^6^ BM-MSCs plus HA. The baseline characteristics of age, body mass index and OA severity, according to the Kellgren–Lawrence (K-L) scale, were well balanced between the groups (Table 1).

### 3.2. Clinical Effects

**Figure 1:**
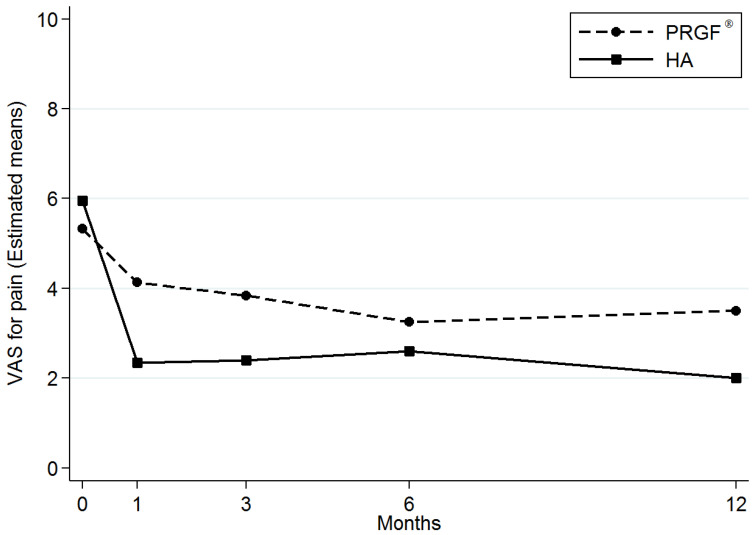
Visual analog scale (VAS) for pain over time according to coadjuvant. HA—hyaluronic acid. PRP—platelet-rich plasma.

The descriptive statistics of VAS for pain over time and according to the coadjuvant group are summarized in Supplementary Table S1. The assessment of pain by VAS showed an improvement in pain when compared with the baseline in both groups (Figure 1). On average, the BM-MSCs plus HA group showed a higher improvement in VAS for pain [β-coefficient: −1.25; 95% confidence interval (95% CI): −2.20 to −0.30) compared with the BM-MSCs plus PRP group (*p* = 0.01) (Table 2, Supplementary Figure S2). The descriptive statistics of WOMAC over time and according to coadjuvant group are detailed in Supplementary Table 2. We also observed differences in all the WOMAC subscale scores and in the WOMAC overall score which were in favor of the treatment using BM-MSCs plus HA compared with the treatment using BM-MSCs plus PRP. However, the differences in WOMAC scores between groups were not statistically significant (Table 3, Supplementary Figure S2).

### 3.3. MRI Findings (WORMS Protocol)

Supplementary Table S3 shows the summary of WORMS over time and according to coadjuvant group. The Whole-Organ Magnetic Resonance Imaging Score (WORMS) at 12 months appeared to be more beneficial in the 100 × 10^6^ BM-MSCs plus HA group (β-coefficient: −12.61; 95% CI: −19.71, −5.52) when compared with the BM-MSC plus PRP group (*p* = 0.001) (Table 4, Supplementary Figure S2).

## 4. Discussion

In this study, we observed that the intra-articular injection of BM-MSCs with different coadjuvants influences the clinical and radiological outcomes in the treatment of knee OA. These results correlate with what is observed in vitro, in which the cell culture medium influences MSCs’ phenotype [6].

In OA, several studies have found that a pain scale Patient Acceptable Symptom State (PASS) value of 4.0 in the VAS can be used as an outcome criterion [26,27]. As previously published in the two clinical trials, the level of VAS achieved at 12 months was 2 (SD: 1.2) in the BM-MSCs plus HA group and 3.5 (SD: 2.5) in the BM-MSCs plus PRP group [3,5]. In other words, the difference reported here between the groups in the VAS explains why a higher proportion of patients (more than 95%) who received BM-MSCs with HA reached the PASS, even from a slightly worse baseline situation (5.3 (SD:1.9) BM-MSCs plus PRP group vs. 5.95 (SD: 2.9) in BM-MSCs plus HA group) [3,5].

It has been reported that, depending on the intensity of the stimuli received, BM-MSCs can act either as pro-inflammatory (phenotype MSC-1) or anti-inflammatory agents (phenotype MSC-2) [28]. Under a pro-inflammatory environment, certain toll-like receptor (TLR) ligands stimulate the immunomodulatory properties of BM-MSCs to produce specific phenotypes [29,30,31]. In OA, both TNF-a and IFN-γ could play an important role in the stimulation of BM-MSCs. In early OA, molecules produced by tissue damage and low amounts of TNF-α and IFN-γ stimulate the TLR-2/4, which polarizes BM-MSCs into an MSC-1 phenotype, secreting chemokines such as Macrophage Inflammatory Protein 1a and 1b (MIP-1a, MIP-1b), CCL5 chemokine (RANTES), CXCL9 chemokine, and CXCL10 chemokine, all of which are responsible for recruiting effector T cells, whose objective is the debridement of the injured area and, therefore, tissue protection [32,33]. However, in chronic processes, high concentrations of TNF-α and IFN-γ stimulate TLR-3 receptors by activating the BM-MSCs 2 phenotype. This produces factors such as IDO, PGE_2_, NO, TGF-β, HGF, HLA-G, among others, all capable of suppressing the proliferation of T lymphocytes and stimulating the production of Treg lymphocytes. This sequence of events will prevent prolonged joint damage. Unfortunately, native BM-MSCs at the site of injury are generally not effective enough to restore immune and regenerative homeostasis in the joint and stop the degenerative process in OA patients [33,34].

According to the results of our study, HA could be clinically more effective as an adjuvant than PRP. In addition, although previous publications from our group did not report a true regenerative effect, we observed a lower joint deterioration in the group that received the HA as a coadjuvant [3,5,35]. It has been suggested that the deposition of HA in the synovial membrane, and on the chondrocytes, would improve the homing and adhesion of the BM-MSCs. This greater effect could be explained by the fact that the synovium is where the inflammatory cells, involved in the inflammatory physiology of OA, are present [36]. The interaction between HA and BM-MSCs has been described previously via the CD44 receptor, an abundant transmembrane glycoprotein present in MSCs, which mediates cellular functions such as migration and adhesion [7,22,32,37]. In the same manner, the interaction between HA and CD44 on chondrocytes has been demonstrated to be crucial for the maintenance of cartilage homeostasis [33,34,38,39]. In fact, when the CD44 receptor was blocked, the mRNA expression levels of chondrogenic marker genes, including *SOX9, COL2A1*, and *ACAN*, were dropped, revealing the important role of this receptor in chondrogenic homeostasis [23]. In consequence, it could explain the lower joint deterioration detected in the WORMS protocol using HA as an adjuvant. We believe that this is an important avenue for future research, based on the rationale of BM-MSC are more effective in earlier OA grades on the K-L scale.

Hyaluronic acid alone would not greatly influence the change in the inflammatory intra-articular environment where BM-MSC activity is expected. In the synovial fluid of a joint, various chemokines can be identified, including TNF-α, IFN-γ or IL-6, all of which activate BM-MSC towards an anti-inflammatory phenotype, stimulating the secretion of a large array of cytokines, such as PGE_2_, GM-CSF, IL-1RA, IL-7, IL-8, IL-10 and IL-11, as well as SDF-1 and other growth factors [28,40,41,42]. This activation towards an anti-inflammatory profile could be even greater in OA of a longer duration. We should note that 50% of the patients included in this study were grade IV, according to the K-L classification system.

The application of PRP is expected to improve the homing and retention of BM-MSCs. Some authors have reported a positive effect on joint lubrication through the stimulation of the synoviocytes-derived secretion of hyaluronic acid by PRP; thus, when administered as a coadjuvant of MSCs, PRP could increase the intra-articular retention and survival of MSCs in an anti-inflammatory environment [43,44]. Although this effect did not exhibit the same level observed as when we used HA, we should remark that the coadjuvant administration was performed immediately following cell treatment. Perhaps, changing the order of coadjuvant administration could lead to different results. Further investigations would be necessary.

The use or non-use of coadjuvants with cell therapies is an interesting topic that should be evaluated specifically, but that has not been the objective of this work. In a previous research, we reported that clinical results with 100 million BM-MSCs with HA and 40 million without adjuvant failed to show significant differences [3]. This unexpected result may arise from the saturation of the healing effect and/or from cell damage due to the transport by oxygen and substrate starvation at the high cell densities used (100 million BM-MSCs with HA) and not in the 40 million BM-MSC group. We have preliminary results that suggest a decrease in cell viability during long storage periods at high density, even at 4 °C. Whether the association of 40 million cells with HA could have greater clinical effects than with the isolated use of BM-MSCs is an interesting question that may deserve future research attention. No comparison with the same dose, density and storage condition has been made previously.

Due to the current doubts about MSCs and the high prevalence of osteoarthritis, numerous researchers are constantly looking for new lines of treatment against osteoarthritis [45,46,47].

The present study is not exempt from limitations. First, the source of our data was randomized clinical trials but these evaluations were not double-blinded. To minimize this potential weakness, subjective clinical scores were complemented with objective measures. In addition, independent radiologists involved in the MRI analyses were masked in the treatment allocation in both clinical trials. Second, we compared two groups of patients from two clinical trials with differences in the target interventions (BM-MSCs dose and adjuvant) and in the age range used as inclusion criteria. This may raise the question of whether these two populations were sufficiently comparable. For the current evaluation, the study sample was restricted to patients who received 100 × 10^6^ BM-MSCs plus an adjuvant without basal demographic differences. This allowed us to evaluate patients that received similar doses of BM-MSCs. Therefore, the difference in the intervention between groups was based mainly on the adjuvant (HA or PRP) to BM-MSCs therapy. Both groups were, in fact, quite comparable in terms of age and other factors such as sex, BMI or severity of knee OA at baseline. Third, the final study sample was relatively small. Thus, our findings should be interpreted with caution until replication in a larger sample and/or using a double-blinded randomized design.

## 5. Conclusions

The clinical and radiological outcomes of BM-MSCs in the treatment of knee OA could be different according to the adjuvant to this therapy. HA appeared to be clinically more effective in terms of the VAS for pain, achieving the PASS in a higher proportion of patients, with less articular degeneration, than PRP as an adjuvant.

## Figures and Tables

**Table 1:** Baseline characteristics of the patients.

	100 × 10^6^ BM-MSC + PRP	100 × 10^6^ BM-MSC + HA
*n*	24	10
Age (years)	56.0 (8.4)	58.4 (4.5)
Males; *n* (%)	17 (70.8)	8 (80)
BMI	27.3 (3.1)	28.5 (3.7)
K-L2; *n* (%)	5 (20.8)	2 (20)
K-L3; *n* (%)	7 (29.2)	4 (40)
K-L4; *n* (%)	12 (50)	4 (40)

BMI—body mass index. HA—hyaluronic acid. K-L—Kellgren and Lawrence grading scale of severity of knee osteoarthritis. PRP—platelet-rich plasma. The data are expressed as means (standard deviation), unless otherwise specified.

**Table 2:** Changes in visual analog scale (VAS) for pain according to coadjuvant.

Group	β-Coefficient (95% CI) *	*p*-Value **
100 × 10^6^ BM-MSC + PRP	Reference	-
100 × 10^6^ BM-MSC + HA	−1.25 (−2.20 to −0.30)	0.010

95% CI—95% confidence interval. BM-MSC—bone marrow mesenchymal stem cell. HA—hyaluronic acid. PRP—platelet-rich plasma. * adjusted for baseline visual analog scale for pain. ** Bonferroni corrected *p*-value: 0.029.

**Table 3:** Changes in Western Ontario and McMaster Universities Osteoarthritis index (WOMAC) according to coadjuvant.

WOMAC	Group	β-Coefficient (95% CI) *	*p*-Value **
Pain	100 × 10^6^ BM-MSC + PRP	Reference	-
	100 × 10^6^ BM-MSC + HA	−0.89 (−2.35 to 0.58)	0.236
Stiffness	100 × 10^6^ BM-MSC + PRP	Reference	-
	100 × 10^6^ BM-MSC + HA	−0.20 (−0.93 to 0.54)	0.599
Function	100 × 10^6^ BM-MSC + PRP	Reference	-
	100 × 10^6^ BM-MSC + HA	−2.56 (−7.68 to 2.57)	0.328
Overall	100 × 10^6^ BM-MSC + PRP	Reference	-
	100 × 10^6^ BM-MSC + HA	−3.63 (−10.78 to 3.53)	0.320

95% CI—95% confidence interval. BM-MSC—bone marrow mesenchymal stem cell. HA—hyaluronic acid. PRP—platelet-rich plasma. * adjusted for baseline Western Ontario and McMaster Universities Osteoarthritis index. ** Bonferroni corrected *p*-values for pain, stiffness, function and overall: 0.709, 1.000, 0.983, and 0.961, respectively.

**Table 4:** Changes in Whole-Organ Magnetic Resonance Imaging Score (WORMS) at 12 months according to coadjuvant.

Group	β-Coefficient (95% CI) *	*p*-Value **
100 × 10^6^ BM-MSC + PRP	Reference	-
100 × 10^6^ BM-MSC + HA	−12.61 (−19.71 to −5.52)	0.001

95% CI—95% confidence interval. BM-MSC—bone marrow mesenchymal stem cell. HA—hyaluronic acid. PRP—platelet-rich plasma. * adjusted for baseline Whole-Organ Magnetic Resonance Imaging Score. ** Bonferroni corrected *p*-value: 0.003.

## Data Availability

The original contributions presented in this study are included in the article/Supplementary Materials. Further inquiries can be directed to the corresponding authors.

## Supplementary Materials

The following supporting information can be downloaded at: https://www.mdpi.com/article/10.3390/diagnostics15030309/s1, Figure S1: CONSORT diagram of the patients; Figure S2: Forest plots of coadjuvant effects for visual analog scale for pain, Western Ontario and McMaster Universities Osteoarthritis index, and Whole-Organ Magnetic Resonance Imaging Score; Table S1:Summary of visual analog scale (VAS) for pain over time and according to intervention group; Table S2: Summary of Western Ontario and McMaster Universities Osteoarthritis index (WOMAC) over time and according to intervention group; Table S3: Summary of Whole-Organ Magnetic Resonance Imaging Score (WORMS) according to intervention group. References [3,5] are cited in the Supplementary Materials.
