# Supplementary material for: Effect of Hyaluronic Acid Compared to Platelet-Rich Plasma as Adjuvants to Bone Marrow Mesenchymal Stem Cell Treatment of Knee Osteoarthritis: Analysis from Two Clinical Trials
Source: Diagnostics (Basel). 2025 Jan 28;15(3):309. doi: 10.3390/diagnostics15030309 (PMC11816799; doi:10.3390/diagnostics15030309)
Supplement: Supplementary file 1 [file diagnostics-15-00309-s001.zip › diagnostics-3405968-supplementary.pdf]

Supplementary figures

Supplementary Figure S1. CONSORT diagram of the patients.

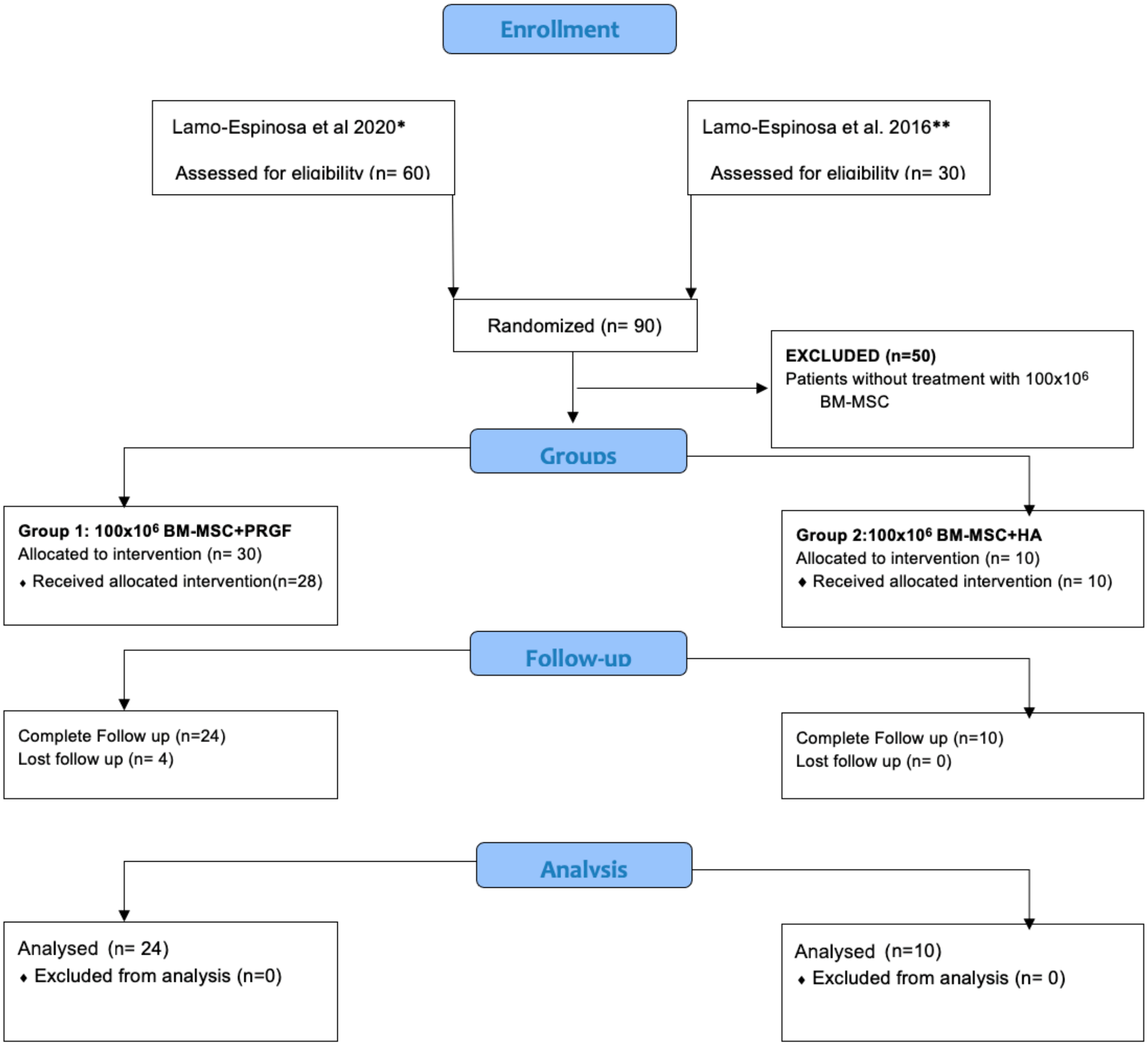

*Data published from Clinical Trial Nº EudraCT: 2011-006036-23, Clinical Trials.gov identifier: NCT02365142 [5]

**Data published from Clinical Trial EudraCT No: 2009-017624-72, Clinical Trials.gov identifier: NCT02123368 [3]

Supplementary Figure S2. Forest plots of coadjuvant effects for visual analog scale for pain, Western Ontario and McMaster Universities Osteoarthritis index, and Whole-Organ Magnetic Resonance Imaging Score.

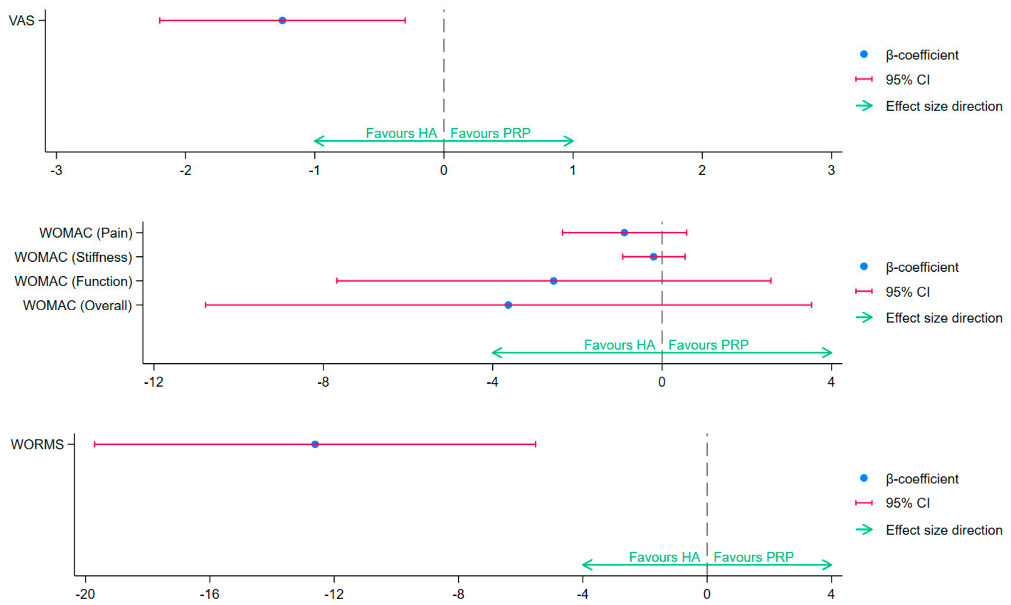

95% CI—95% confidence interval. HA—hyaluronic acid. PRP—platelet-rich plasma. VAS—visual analogue scale for pain. WOMAC—Western Ontario and McMaster Universities Osteoarthritis index. WORMS—Whole-Organ Magnetic Resonance Imaging Score.

Supplementary tables

Supplementary Table S1. Summary of visual analog scale (VAS) for pain over time and according to intervention group.

Time	100 × 106 BM-MSC + PRP	100 × 106 BM-MSC + HA
	Mean (SD)	Mean (SD)
Baseline	5.3 (1.9)	6.0 (2.3)
1 month	4.1 (2.5)	2.4 (1.9)
3 months	3.8 (2.0)	2.4 (1.8)
6 months	3.3 (2.2)	2.6 (2.8)
12 months	3.5 (2.5)	2.0 (1.6)

BM-MSC—bone marrow mesenchymal stem cell. HA—hyaluronic acid. PRP—platelet-rich plasma. SD—standard deviation.

Supplementary Table S2. Summary of Western Ontario and McMaster Universities Osteoarthritis index (WOMAC) over time and according to intervention group.

WOMAC	Time	100 × 106 BM-MSC + PRP	100 × 106 BM-MSC + HA
		Mean (SD)	Mean (SD)
Pain	Baseline	6.6 (4.4)	4.7 (2.1)
	1 month	5.7 (3.8)	3.0 (2.3)
	3 months	4.6 (3.3)	3.5 (2.2)
	6 months	4.3 (3.6)	3.7 (2.5)
	12 months	4.1 (3.6)	2.5 (1.4)
Stiffness	Baseline	3.3 (2.1)	2.7 (1.1)

	1 month	2.6 (1.6)	2.0 (1.2)
	3 months	2.3 (2.2)	1.9 (1.4)
	6 months	2.0 (1.9)	2.0 (1.5)
	12 months	2.1 (1.9)	1.5 (0.7)
Physical function	Baseline	23.5 (13.2)	18.9 (7.6)
	1 month	19.8 (12.4)	13.0 (8.7)
	3 months	17.6 (12.6)	12.3 (9.3)
	6 months	14.9 (11.8)	14.8 (11.4)
	12 months	16.7 (11.6)	10.5 (4.7)
Overall	Baseline	33.4 (18.7)	26.3 (8.9)
	1 month	28.1 (16.7)	18.0 (11.4)
	3 months	24.4 (17.4)	17.7 (12.4)
	6 months	21.3 (16.6)	20.5 (14.6)
	12 months	23.0 (16.6)	14.5 (5.6)

BM-MSC—bone marrow mesenchymal stem cell. HA—hyaluronic acid. PRP—platelet-rich plasma. SD—standard deviation.

Supplementary Table S3. Summary of Whole-Organ Magnetic Resonance Imaging Score (WORMS) according to intervention group.

Time	100 × 106 BM-MSC + PRP	100 × 106 BM-MSC + HA
	Mean (SD)	Mean (SD)
Baseline	74.4 (28.0)	68.6 (24.3)
12 months	79.8 (29.1)	61.4 (26.7)

BM-MSC—bone marrow mesenchymal stem cell. HA—hyaluronic acid. PRP—platelet-rich plasma. SD—standard deviation.
